# Blunted cardiac autonomic dynamics to active standing test in postmenopausal women

**DOI:** 10.3389/fcvm.2024.1402086

**Published:** 2024-08-20

**Authors:** Costanza Scatà, Felipe C. Ferreira, Michelle C. S. V. Padilha, Angelica Carandina, Riccardo Asnaghi, Chiara Bellocchi, Eleonora Tobaldini, Nicola Montano, Pedro Paulo da S. Soares, Gabriel D. Rodrigues

**Affiliations:** ^1^ Department of Internal Medicine, Fondazione IRCCS Ca’ Granda Ospedale Maggiore Policlinico, Milan, Italy; ^2^ Department of Physiology and Pharmacology, Fluminense Federal University, Niterói, Brazil; ^3^ Department of Clinical Sciences and Community Health, University of Milan, Milan, Italy

**Keywords:** heart rate variability, orthostatic stress, menopause, estrogen, aging

## Abstract

**Introduction:**

Although both aging and menopause influence cardiovascular autonomic control, the effect of menopause *per se* remains unclear. The current study was undertaken to test the hypothesis that post-menopausal women (PMW) have a blunted cardiovascular autonomic adjustment to active standing compared to pre-menopausal women. Thus, we compared the heart rate variability (HRV) indexes from supine (SUP) to orthostatic (ORT) positions among young women (YW), young men (YM), older men (OM), and PMW.

**Methods:**

The participants rested for 10 min in SUP and then stood up and remained for 5 min in ORT. ECG was continuously recorded, and R-R time series of about 300 beats were analyzed using linear (spectral analysis) and non-linear (symbolic analysis) methods. The variation from SUP to ORT was calculated (*Δ* = ORT-SUP) for each HRV index.

**Results:**

In SUP, no difference was found for any HRV index among groups. However, *Δ*0V% and *Δ*LFn (cardiac sympathetic modulation) were reduced in PWM compared to all groups (OM, YW, and YM), while *Δ*2UV% and *Δ*HFn (cardiac vagal modulation) were reduced in PMW than the younger group (YW and YM). No differences were found among the male groups (OM and YM).

**Discussion:**

In light of our results, the cardiac autonomic dynamic response to orthostatic stress is blunted in post-menopausal women compared to younger women and older men, a finding that might be influenced not only by aging.

## Introduction

With aging, ovarian follicles diminish in number, and, consequently, there is a decrease in inhibin and estrogen production that leads to a failure of endometrial development and anovulation (1). The age of the final menstruation period generally varies between 40 and 58 years (1, 2). Menopausal symptoms include genitourinary (3) and depressive symptoms, such as loss of interest and energy or ideas or attempts at self-harm (4, 5), cognitive impairments (6), sleep difficulties (7), and vasomotor symptoms [e.g., hot flushes and night sweats; (8)]. These symptoms are related to the hormonal alterations involved in this process and negatively affect women's quality of life (9).

Pre-menopausal women have a lower risk of developing coronary vascular disease (CVD) compared to men of the same age (10). However, the sex gap in cardiovascular morbidity and mortality tends to diminish after the age of 45 (2). Indeed, increased levels of bioavailable testosterone (7) and declining estrogen levels (11) could lead to increased risk factors, such as a change in the composition and distribution of abdominal visceral fat or lipid deposition around the heart and aorta (12), altered insulin action [in terms of insulin sensitivity and glucose tolerance; (13, 14)], and hypertension (15), all of which are related to increased cardiovascular morbidity (16). Finally, another factor that can influence the loss of cardioprotection in post-menopause women is increased arterial stiffness, due to the loss of the beneficial vasoactive effect of estrogens (17, 18).

Another factor that may account for the increased cardiovascular risk of postmenopausal women, and which interacts closely with the previous ones, includes impairments in cardiovascular autonomic control (CAC). Estrogens have an independent favorable modulatory effect on the autonomic nervous system (ANS) (19), and pre-menopausal women have higher cardiac vagal tone compared to men of the same age (20, 21), which has been positively correlated with estradiol levels (22). In contrast, an estrogen deficiency due to natural or surgical menopause is associated with a shift toward decreased cardiac vagal control and/or increased sympathetic tone (23, 24) leading to reduced Heart Rate Variability (HRV) (25), which has been associated with increased cardiovascular mortality and morbidity (26, 27).

HRV is a non-invasive tool that informs about sympatho-vagal interactions at the cardiac level in response to internal and external stressors (27, 28). Physiologically, parasympathetic nervous system (PNS) activity prevails during resting conditions, while the sympathetic nervous system (SNS) is mainly activated during triggering situations (29). Methodologically, this balance is assessed by changes in the interval between one beat and the subsequent one [known as the R-R interval, referring to the QRS complex; (30)]. The more variable the system is (namely, having a high value of HRV), the better it responds to different stimulations and is associated with good cardiovascular health (31).

A seminar study by Montano and colleagues (28) described a reduction in cardiac parasympathetic while sympathetic modulation increases along with the body inclination during the tilt test. In addition, cardiovascular autonomic response to orthostatic stress is impaired in aging (32, 33), cardiopulmonary (34) autoimmune (35), and neurodegenerative (36) diseases. Also, nonlinear HRV methods add a better physiological and clinical interpretation since some patterns were not observed while employing only linear HRV analysis (34–36). In this perspective, combining both HRV linear and nonlinear methods at rest and during active standing tests has been a suitable setup to explore the cardiovascular autonomic dynamics across the lifespan (33, 35, 37).

The current study was undertaken to test the hypothesis that PMW has an attenuated cardiovascular autonomic adjustment to orthostatic postural challenge compared to pre-menopausal women and male controls. To do so, the study aimed to compare HRV changes from the supine position to active standing among young women (YW), young men (YM), older men (OM), and PMW.

## Methods

### Dataset

Pre-menopausal and post-menopausal women and their age-matched male controls participated in the current study. Forty-eight healthy (*n* = 12 per group), non-diabetic, non-smoking, and free of cardiovascular diseases participants were enrolled in the current study. None of them was on any cardiovascular-acting medications or any medications that could influence the outcomes. All participants included in the current study were recreationally physically active (self-reported). YW group was evaluated during the early follicular phase, counted as the first day of the menstrual cycle until the 6th day, as informed by the participants, and users of any hormonal contraception method were excluded. PMW was defined as at least one year after the last menstruation (38), and PMW under menopausal hormone therapy was excluded.

The sample size was calculated to ensure a statistical power of 0.80 or higher for the main outcome variables *Δ*2UV% and *Δ*HFn. The enrolled sample provided a statistical power of 1.00 and 0.96 for these variables, respectively. The sample size (*n*) is determined based on the required power level (1 − *β*), the significance level (*α* < 0.05), and the effect size to be detected with a probability of 1 − *β*. For the calculation of sample power, we used the parameters *α* < 0.05, number of groups (4), number of cofactors (1), and effect size (calculated by partial *η*2) (39). Consequently, for the main outcome variables *Δ*2UV% and *Δ*HFn, we conducted a study with 12 volunteers in each group. After affirming that the statistical power was greater than 0.8, we conducted a *post hoc* analysis to confirm these assumptions.

The institutional ethics committee from Fluminense Federal University (#4.534.903), according to the Declaration of Helsinki, approved the procedures, and all volunteers provided informed consent in written form before participating in the study.

### Experimental protocol

The experimental protocol was performed in a single day. Participants were familiarized with all study procedures, instructed to avoid strenuous physical activity [≥5 METs; according to (40)] on the day before testing, to have the last meal no later than 2 h preceding the test, and not to ingest caffeine, alcohol, or another substance that could influence the study outcome. Anthropometric measures were undertaken of the height (assessed using a portable stadiometer. Sanny, Brazil) and body mass (BM) (through an electronic scale with 10 g of precision. W-200, WELMY, Brazil). After instrumentation procedures, the participants rested for 10 min in the supine position (SUP) and were instructed to stand up for 5 min in the orthostatic position (ORT). The participants breathed spontaneously during the protocol, and the room temperature was controlled at 22–24°C. R-R signals were continuously recorded during the experimental protocol.

### Cardiovascular autonomic control assessment

From the ECG signals, the apex of the R wave was located using parabolic interpolation to obtain the tachogram, trend over time of the R-R series. The R-R is the elapsed time (interval) between different R-wave peaks on the QRS complex). R-R artifact-free time series of at least 300 beats in SUP and ORT were used in two different approaches of HRV analysis: linear (time and frequency domains) and non-linear analysis.

The time domain indexes included the mean of the standard deviations of all R-R intervals (SDNN) and the root mean squared difference of successive R-R intervals (RMSSD), which was considered indexes of global variability and vagal modulation (41). The time domain HRV were obtained using the LabChart-Pro8 (ADInstruments, Sydney Australia).

For spectral analysis, the autoregressive model was performed to extract the rhythmic oscillations that characterize the R-R time series. The Total Power, which represents the global autonomic modulation to the cardiovascular system, and three main components of the total spectrum can be identified: the spectral power in the very low-frequency band (VLF, frequency band below 0.04 Hz), the spectral power in the low frequency (LF, frequency band bounded between 0.04 and 0.15 Hz), index of both sympathetic and vagal modulations, and the spectral power in the high frequency (HF, frequency band bounded between 0.15 and 0.40 Hz), index of parasympathetic modulation and synchronous with respiratory activity. The model order to identify the frequency bands was estimated for each segment by Akaike information criterion ranging from 5 to 14. The powers of LF and HF components were expressed in normalized units LF (n.u) and HF (n.u) dividing each band power by the total power subtracted by the VLF component (<0.04 Hz) (28, 35).

Nonlinear analysis was performed using symbolic dynamics on the same segments identified for spectral analysis. To perform symbolic analysis, the R-R time series was converted into a sequence of symbols that was divided into 3-beat patterns. Patterns were classified into 4 families: (a) 0 V, patterns with no variation, all 3 symbols are equal (e.g., 4-4-4); (b) 1 V, patterns with 1 variation, 2 consecutive symbols are equal forming a 2-beat plateau, while the remaining one is different (e.g., 2-2-5); (c) 2LV, patterns with 2 like variations, all symbols are different from the previous one and they are in ascending or descending order (e.g., 1-3-4); (d) 2UV, patterns with 2 unlike variations, all symbols are different from the previous one but not in a consequent order (e.g., 2-5-1). The percentage of the patterns 0 V is a marker of cardiac sympathetic modulation and 2UV or 2LV are markers of cardiac vagal modulation (42). This approach allows for assessing non-reciprocal changes in sympathetic and parasympathetic modulation on heart period time series in physiological and pathological conditions (42, 43). Spectral and symbolic analyses were run through *ad hoc* software (*HeartScope* II AMPS, ITA).

### Statistics

The normality data distribution was evaluated by the Shapiro-Wilk test. The One-way analysis of variance (ANOVA) with Tukey's post-hoc test was used for individual characteristics comparisons and to evaluate the isolated effect of autonomic at rest and dynamic response to ORT (*Δ* = ORT position–SUP position) between groups (37). The analysis of covariance (ANCOVA) was employed to correct the BMI as a cofactor among groups in all comparisons (e.g., HRV in SUP and *Δ*ORT) because BMI has been reported to influence the HRV (44). Also, to correct an influence of baseline HRV in the interpretation of *Δ*ORT, when an HRV index was different among the groups in the supine position it was considered as a cofactor in the *Δ*ORT comparisons.

Statistical significance was assumed for an alpha error probability <0.05. The software used for statistics was SPSS Statistics version 21.0 (IBM Corp., Armonk, NY, USA), graphs were built in GraphPad Prism version 10.0 (GraphPad Software Inc., San Diego, CA, USA), and the statistical power was calculated using the G-Power version 3.1.9.2 (Heinrich-Heine-Universit¨at Düsseldorf, Düsseldorf, Germany).

## Results

### Participant's characteristics

There were sex differences in body mass, which was higher in OM than PMW and YW, and higher in YM than YW. OM and YM presented a higher stature than YW and PMW. PMW and OM presented a higher BMI than YW and YM. All characteristics, comorbidities, and medications are presented in Table 1.

**Table 1:** General characteristics, comorbidities and medications of participants.

	PMW	OM	YW	YM	*p*-value
General characteristics					
*N*	12	12	12	12	–
Aged (years)	58 ± 3	59 ± 3	27 ± 3^a^^,^^b^	27 ± 4^a^^,^^b^	<0.01
Stature (cm)	156 ± 6	173 ± 7^a^^,^^b^^,^^c^	165 ± 4	175 ± 5^a^^,^^b^^,^^c^	<0.01
Body mass (kg)	66 ± 9	77 ± 10^a^^,^^c^	60 ± 5	73 ± 6^c^	<0.01
BMI (kg/m^2^)	27 ± 2	26 ± 3	22 ± 2^a^^,^^b^	24 ± 1^a^^,^^b^	<0.01
Comorbidities					
Osteoporosis (*n*/%)	6 (50%)	2 (16%)	0	0	–
Gastrointestinal disorders (*n*/%)	4 (33%)	5 (41%)	0	0	–
Hypothyroidism (*n*/%)	2 (16%)	0	0	0	–
Hormone therapy	0	0	0	0	–
Medications					
Calcium supplements	4 (33%)	0	0	0	–
Proton-pump inhibitors	4 (33%)	5 (41%)	0	0	–
L-thyroxine	2 (16%)	0	0	0	–

Mean ± SD.

PMW, post-menopause women; OM, older man; YW, young women; YM, young men; BMI, body mass index.

One-way ANOVA with the Tukey’s post-hoc test was used. *p* < 0.05.

^a^ Differences from PMW.

^b^ Differences from OM.

^c^ Differences from YW.

### Cardiac autonomic modulation

**Figure 1:**
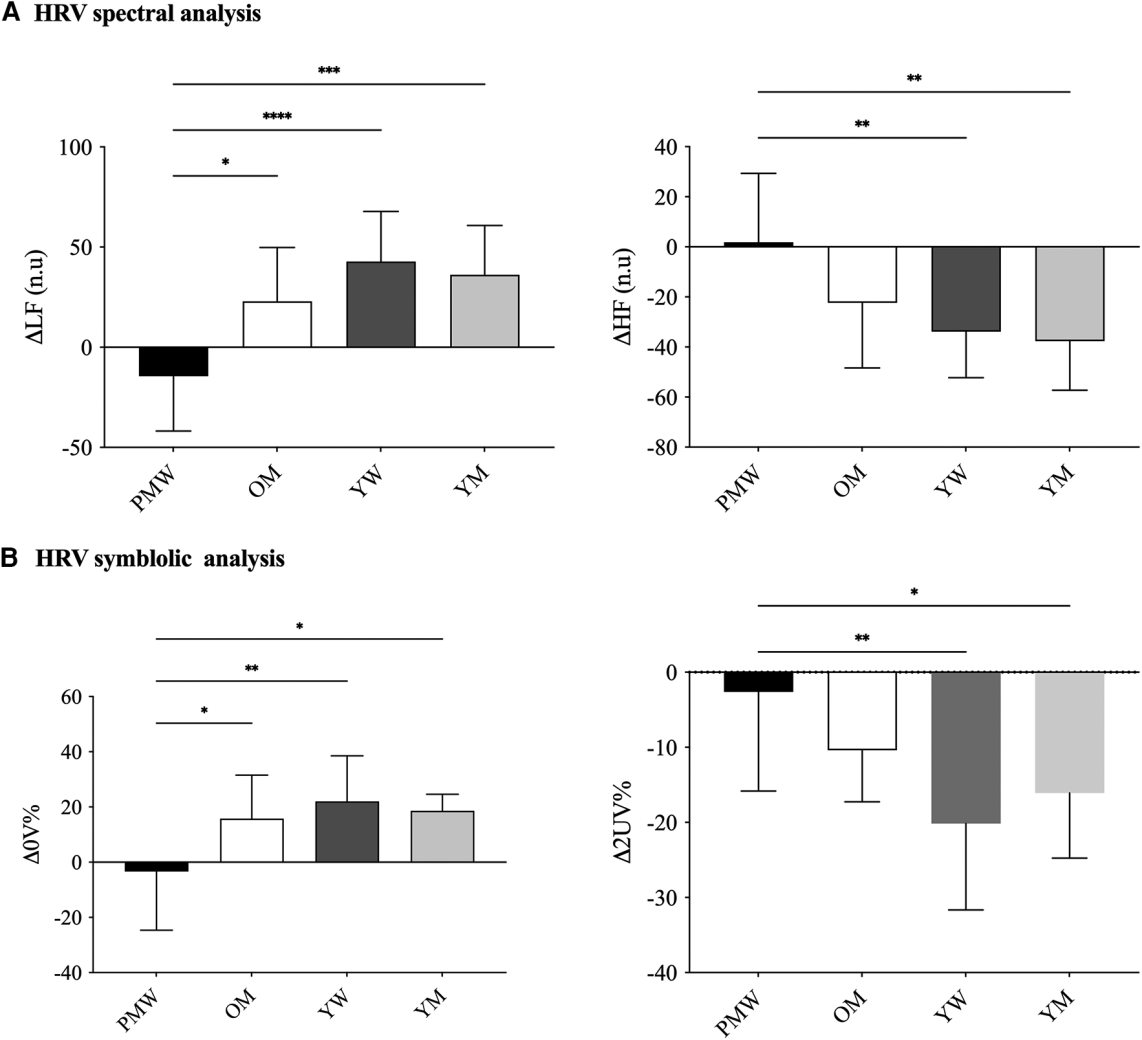
Cardiac autonomic dynamic response to active standing test among groups. Panel **A** represents HRV linear analysis by spectral indexes; Panel **B** represents HRV non-linear analysis by symbolic indexes. PMW, post-menopausal women; OM, older men; YW, young women; YM, young men; LF, low frequency in normalized unity; HF, high frequency in normalized unity; 0 V, patterns with no variations of R-R intervals (sympathetic modulation); 2UV, patterns with two like variations of R-R intervals (vagal modulation); *Δ *= (HRV in orthostatic position—HRV in supine position); Data presented in mean ± SD. The analysis of covariance (ANCOVA) was utilized considering the BMI (24.07 kg/m^2^). *p < 0.05 and **p < 0.01 were reported. Twelve participants in each group were considered in the analyses.

In SUP, HRV total power was reduced in OM and PMW compared to YM and YW (*p* < 0.05). However, there were no differences for other spectral or symbolic HRV indexes among groups (*p* > 0.05). Regarding the HRV time domain, SDNN and RMSSD were reduced in OM and PMW compared to YM and YW (*p* < 0.05; Table 2). In response to ORT, both spectral and symbolic HRV were reduced in PMW. Namely, *Δ*0V% and *Δ*LFn were reduced in PMW compared to all groups (OM, YW, and YM), while *Δ*2UV% and *Δ*HFn (cardiac vagal modulation) were reduced in PMW than the younger group (YW and YM) (Figure 1).

**Table 2:** Cardiac autonomic modulation at rest among groups.

	PMW	OM	YW	YM	*p*-value
HR (bpm)	68 ± 10	64 ± 8	65 ± 9	62 ± 6	0.85
HRV time-domains					
SDNN (ms)	38 ± 28	41 ± 11	66 ± 15^a^^,^^b^	68 ± 16^a^^,^^b^	0.01
rMSSD (ms)	32 ± 20	31 ± 10	65 ± 24^a^^,^^b^	72 ± 24^a^^,^^b^	0.01
HRV spectral analysis					
Total power (ms^2^)	2,401 ± 3,760	1,953 ± 763	4,102^a^^,^^b^^ ^± 2,587	4,870^a^^,^^b^ ± 3,306	0.02
LF (n.u)	53 ± 16	64 ± 24	43 ± 24	44 ± 19	0.35
HF (n.u)	45 ± 18	32 ± 18	45 ± 20	45 ± 21	0.61
HRV symbolic analysis					
0 V (%)	23 ± 16	27 ± 13	15 ± 8	15 ± 8	0.38
2UV (%)	18 ± 6	16 ± 7	25 ± 10	24 ± 10	0.18
2LV (%)	10 ± 7	8 ± 3	15 ± 12	15 ± 11	0.86

Mean ± SD.

PMW, post-menopausal women; OM, older men; YW, young women; YM, young men; LF, low frequency in normalized unity; HF, high frequency in normalized unity; LF/HF, sympatho-vagal balance; 0 V, patterns with no variations of R-R intervals (sympathetic modulation); 2UV, patterns with two like variations of R-R intervals (vagal modulation); 2LV, patterns with two unlike variations of R-R intervals (vagal modulation); SDNN, mean of the standard deviations of all R-R intervals; RMSSD, root square of the mean of the squares of successive differences between adjacent R-R intervals.

The analysis of covariance (ANCOVA) was considered the BMI: 24.07 kg/m^2^. *p* < 0.05. Twelve participants in each group were included in the analyses.

No differences were found among the male groups (OM and YM). HRV in time-domain *Δ*RMSSD varied more negatively in YW and YM compared to older groups (YW −38.1 ± 30.1; YM −27.7 ± 23.1; PMW −6.7 ± 15.0; OM −13.5 ± 6.1 ms; *p* = 0.02), without sex-differences. However, no differences for *Δ*SDNN (YW −17.1 ± 20.7; YM −6.1 ± 11.7; PMW −8.3 ± 20.7; OM −7.1 ± 17.8 ms; *p* = 0.32) were found among groups. The BMI was employed as a covariate in all comparisons. The absence of differences for *Δ*SDNN and *Δ*Total Power indexes was confirmed when their respective values in SUP position were included in ANCOVA. Differences in *Δ*RMSSD remained even after considering the baseline (i.e., ANCOVA using the RMSSD at rest as a covariable).

## Discussion

The current study reveals that post-menopausal women exhibit a blunted cardiac autonomic response to active standing in comparison to younger women, men, and older men. Secondly, the lack of differences between older men and the younger groups (men and women) indicates that the findings are related to menopause alone rather than being a result of aging.

Previous studies suggested that pre-menopausal women have higher cardiac vagal tone than age-matched men (20, 21). Estrogens seem to be a putative mechanism underlying cardiac vagal autonomic upregulation observed in young women (19, 22). On the other hand, an acute ovarian hormone retreat induced by oophorectomy shifted the sympatho-vagal balance towards a sympathetic predominance and vagal withdrawal (i.e., decreased vagal tone). However, a group of women who underwent a hysterectomy were included in the study to rule out the possibility that any changes in their cardiac autonomic function were not due solely to the surgical procedure or its psychological effects (23).

Both animal and human studies indicate a higher vagal-mediated response to coronary artery occlusion in females than in males (45, 46). It might be justified, at least in part, by estrogen, since after ovariectomy the vagal activity in females was closer to that of males (47). In rodents, the sympatho-vagal balance increased after ovariectomy compared to sham surgery, supporting the possible cardioprotective role of ovarian hormones (48). Another study found that ovariectomy did not influence the sympatho-vagal balance in spontaneously hypertensive rats (SHR) or Wistar rats (controls). However, estradiol therapy increased the sympathetic modulation to the heart in both SHR and controls (49). Also, oxytocin plays a role in autonomic nervous control, stimulating oxytocin neurons to increase vagal tone and reduce heart rate (50, 51). A recent meta-analysis aimed to investigate the role of estrogen concentrations in cardiac autonomic regulation in women with migraine. Although reduced estrogen concentrations during the luteal phase of the menstrual cycle, no difference was found in HRV indexes compared to controls (52). Indeed, normal cyclical variations in endogenous sex hormone levels throughout the menstrual cycle (menstruation, ovulation, and luteal) were not associated with any change in cardiac autonomic control (53). In this way, the potential sex differences in cardiac autonomic control across the lifespan might be explained by a combination of humoral and neural control mechanisms, which should be considered in further HRV studies.

Natural menopause leads to a similar effect to the oophorectomy study (23), increasing the sympathetic tone (54, 55) and leading to vagal withdrawal (56, 57). A cross-sectional study observed a reduced cardiac vagal modulation in PMW compared to pre-menopausal women. However, estradiol level and HRV indexes were not correlated. Thus, the authors suggested that aging should be the main factor of reduced cardiac autonomic modulation in PMW (57). In contrast, the exaggerated increases in muscle sympathetic nervous activity and blood pressure during isometric exercise observed in PMW were attenuated by an acute estradiol administration, suggesting that estradiol mediates sympathetic overactivity during exercise in this population (55). Regarding their limitations, these studies (55, 57) did not compare the PMW with an age-matched group of males, limiting their findings.

To our knowledge, a single study investigated the cardiac autonomic control during postural challenge (supine-to-sitting) in PMW compared to young women and older men (56). Saeki and colleagues assessed the HRV among young women, post-menopausal women, and older men. The study did not include a group of young men matched by age with the young women, which could be seen as a limitation, as it is expected that there could be differences in HRV between young males and females (20). In addition, they employed the supine-to-sitting maneuver, which, regarding cardiac autonomic adjustment, provoked a quieter response when compared to the supine-to-standing test (58). Thus, a slight variation of cardiac autonomic modulation from supine to sitting is expected (58), which may have underestimated their results (56). However, in our study, the cardiac autonomic adjustments during supine-to-stand indicated further information regarding the resting HRV alone in group comparisons, confirming what occurred in several clinical populations (34–36, 59). Lastly, Saeki and colleagues employed only HRV linear methods (i.e., time domain and spectral analysis) to compare the groups. HRV linear methods require a premise of signal stationarity that does not happen in the biological data (42, 43). Previous studies indicated that using nonlinear HRV methods provides a more comprehensive understanding of cardiac autonomic regulation compared to linear HRV analysis alone (34, 35, 42, 43).

The global HRV in the time (SDNN) and frequency (total power) domains and the vagal-mediated HRV in the time domain (RMSSD) are reduced in the older when compared to the younger group, without sex differences. Previous studies (60, 61) showed an age dependence in HRV indexes at rest. However, the differences considering sex and age occurred only in response to ORT stress (Figure 1), suggesting a blunted cardiac autonomic response in PWM compared to the others (YW, YM, and OM). In particular, acute reductions in cardiac vagal modulation were observed during hot flashes in late peri-menopausal and post-menopausal women compared with the periods before and after hot flashes (62). Curiously, pre-menopausal women had greater cardiac vagal withdrawal during acute heat stress exposition compared to men of the same age (21). On the other hand, pretreatment of hot flashes using hormone therapy affected cardiac autonomic control despite an effective reduction in vasomotor hot flashes and an increment in estrogen levels (63).

Our findings suggest that cardiac autonomic control plays a significant role in women's physiology. Also, HRV studies should consider the application of autonomic maneuvers in their setups. Indeed, active standing tests revealed cardiac autonomic patterns that were not observed during rest only (34, 37, 64). Because HRV indexes are associated with an increased risk of cardiovascular events (26, 27), these indexes should be incorporated into healthspan programs. Also, further studies should investigate non-pharmacological strategies to modulate cardiac autonomic control in post-menopausal women.

The current study has some limitations that need to be addressed. The sample size is relatively small. Although menopause was self-reported by the participants, no hormone measurements were taken. We excluded hormonal contraception users and assessed all young women in the same phase of the menstrual cycle, trying to eliminate a potential variation in hormonal status among young women. Also, the groups under study differ in terms of body mass and stature. Nevertheless, BMI was included as a cofactor in this study. Lastly, no other assessments were conducted to examine the differences in muscle, bone, and fat mass. These limitations should be taken into account when interpreting the results of the study.

## Conclusion

Menopause blunts the cardiac autonomic dynamic response to active standing. Since young and older men are not different, aging may not be the only factor influencing this response in post-menopausal women. Further studies investigating putative mechanisms underlying this blunted response are needed to confirm our findings.

## Data Availability

The raw data supporting the conclusions of this article will be made available by the authors, without undue reservation.
